# Favorable long-term outcome in young adults undergoing surgery for lumbar disc herniation

**DOI:** 10.1007/s00701-022-05375-8

**Published:** 2022-10-07

**Authors:** Miika Ilvesroiha, Johan Marjamaa, Jari Siironen, Anniina Koski-Palkén

**Affiliations:** 1 Department of Neurosurgery, Helsinki University Hospital, Helsinki, Finland; 2 Faculty of Medicine, University of Helsinki, Helsinki, Finland

**Keywords:** Lumbar disc herniation, Surgery, Long-term outcome, Oswestry Disability Index

## Abstract

**Purpose:**

The purpose of the study was to evaluate the long-term outcome after surgery for lumbar disc herniation in a young adult population.

**Methods:**

A total of 526 consecutive patients between 18 and 40 years of age who underwent surgery for lumbar disc between 1990 and 2005 were included in the study. The primary outcomes were the need for new lumbar spine surgery during the follow-up and secondary outcomes were short-term subjective outcome, the Oswestry Disability Index (ODI) score, and the ability to carry out employment at the end of the long-term follow-up.

**Results:**

A total of 96% of the patients had a reduction in their symptoms at the clinical follow-up (median of 50 days post-surgery). Twenty-one patients (4.0%) had a reoperation within 28 days. Excluding these early reoperations, 136 patients (26%) had additional lumbar spine surgery and 18 patients (3.4%) underwent lumbar fusion during the follow-up of median 18 years. The annual risk for new surgery was 1.4%. In total, 316 patients (60%) returned the ODI questionnaire, and their mean score was 8.1. Patients with a higher number of additional lumbar spine surgeries (*p* < 0.001) reported deteriorating ODI scores.

**Conclusion:**

Patients showed excellent short-term recovery from their symptoms. In the long term, the mean ODI score for the patients was comparable to the normative population. However, a notable proportion of the patients required additional lumbar surgery during the follow-up period, and a higher number of lumbar surgeries was associated with poor ODI scores.

## Introduction

Sciatica is a common cause of disability, with an estimated prevalence ranging from 1.4 up to 42.3% [9]. If sciatica is severe enough to warrant surgical evaluation, the most common reason for the sciatica is lumbar disc herniation [14]. In these cases, the prognosis is favorable, and most patients experience a significant relief in their symptoms spontaneous within weeks to months [17–19]. Surgery is indicated in cases with either prolonged or primarily unbearable pain episodes or severe neurologic symptoms.

Compared to conservative treatment, surgical treatment has been shown to relieve symptoms within a shorter period of time [1, 20] and allow for a faster return to work [16]. However, the advantage of surgical treatment with regard to outcome diminishes as time passes and the outcome is similar for both treatment groups after 6 months [13].

The current literature does not sufficiently describe the outcomes for patients in their 20 s or 30 s. This is because the rate of occurrence of symptomatic lumbar disc herniation is the highest for those in their early 40 s [3]. Accordingly, several large studies describing the long-term outcome for surgically treated patients have a mean age of over 40 for the patient cohort [1, 21].

The long-term outcome is of particular interest in younger populations since younger adult patients still have most of their working life ahead of them; thus, back-related disability can have major consequences on their quality of life and overall health, and it may also have serious economic consequences for the patients.

We carried out a cohort study on long-term outcomes after surgery for lumbar disc herniation in Finnish adults between the ages of 18 and 40 years. The study included retrospective data collection from medical records and the assessment of patient outcomes at the present time with questionnaires. The primary outcome measures of the study were the need for new lumbar spine surgery and secondary outcomes were short-term subjective outcome, the Oswestry Disability Index (ODI) [4, 12] score, and the ability to carry out employment at the end of the long-term follow-up.

## Methods

### Patient selection

**Fig. 1:**
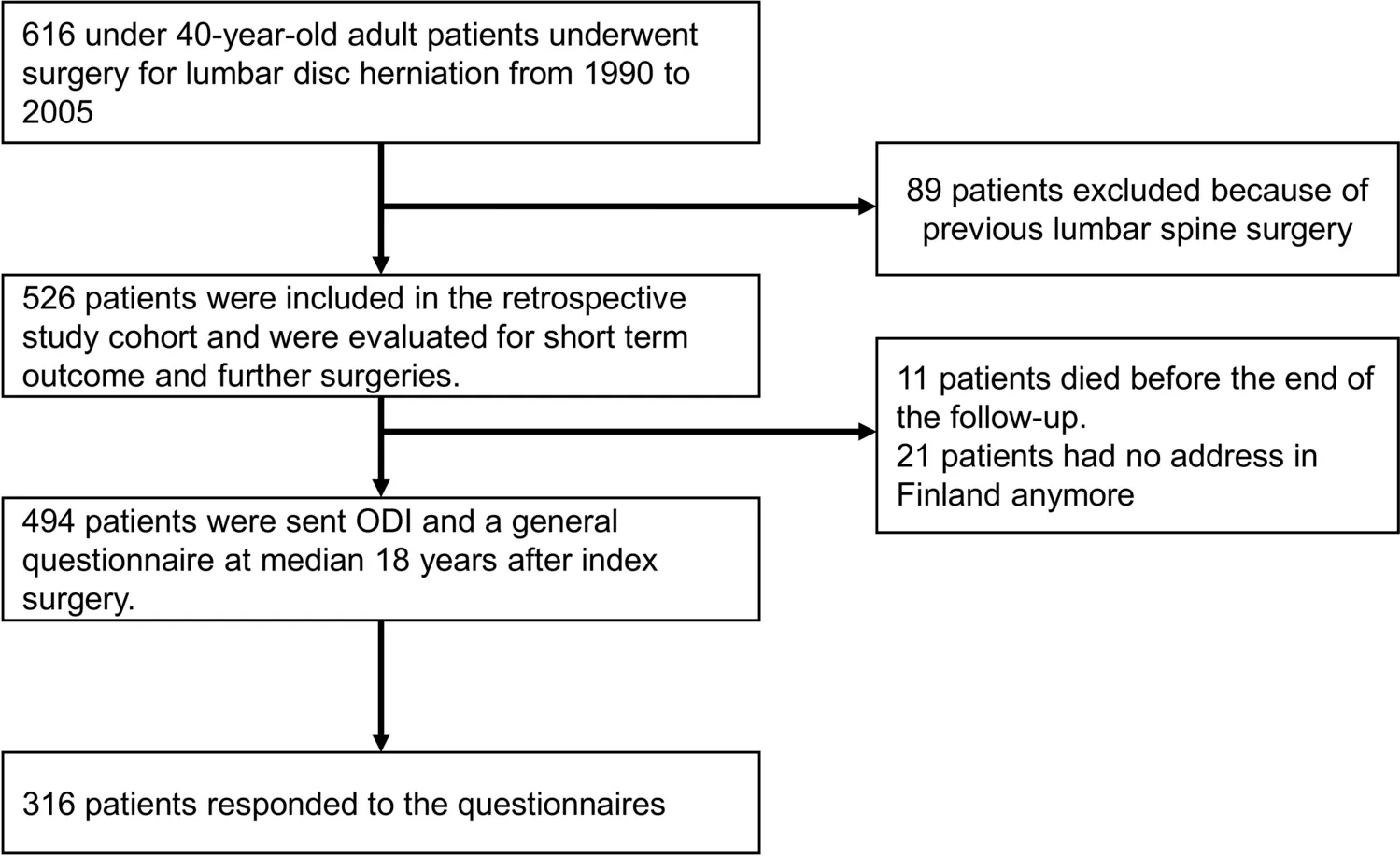
The data collection protocol. In total 616 under 40-year-old adults underwent surgery for lumbar disc herniation at the Department of Neurosurgery at Helsinki University Hospital in Finland. The medical records of these patients were examined, and 89 patients with previous lumbar surgery were excluded. The medical records were used to collect preoperative data, surgical data, and follow-up data. The patients who were still alive and living in Finland were sent Oswestry Disability Index-questionnaire and a general questionnaire which collected information on smoking habits, current employment status, and satisfaction with index surgery. A total of 316 patients responded to the questionnaires

The patient cohort included 526 consecutive 18 to 40-year-old patients who underwent surgery for radiologically confirmed lumbar disc herniation from 1990 to 2005 at the Department of Neurosurgery at Helsinki University Hospital in Finland. Patients were identified based on (ICD-9 and thereafter ICD-10) diagnosis code for lumbar disc herniation as the indication for surgery from the surgical record data base of the hospital. Patients with prior lumbar surgery and patients whose symptoms were caused by entities other than lumbar disc herniation, such as trauma or tumor, were excluded after review of medical records. The routine radiological pre-surgical evaluation was magnetic resonance imaging (MRI). This retrospective study protocol was approved by the ethics committee of Helsinki University Hospital. The patient selection and data collection protocol are presented in Fig. 1.

### Patient cohort

The total number of patients included in the sample was 526. The median age was 33.2 years, and 31% of patients were under 30 years of age at the time of the index surgery. The mean body mass index (BMI) was 24.5, and 36% of the patients were smokers at the time of the surgery. The median follow-up time for the cohort was 18.3 years (Table 1).

**Table 1:** Patient baseline characteristics

	Number (%)
Number of patients	526
Age (years, median, IQR)	33.2 (7.6)
Under 30 years old	165 (31%)
Gender	
Male	314 (60%)
Female	212 (40%)
Smoker^1^	134 (36%)
Body mass index^2^	
Average (SD)	24.5 (3.6)
< 18.5	6 (1.2%)
18.5–24.9	292 (58%)
25–30	172 (34%)
30 +	31 (6.2%)
Duration of symptoms preoperatively	
< 1 month	14 (2.7%)
1–3 months	119 (23%)
3–6 months	159 (30%)
6–12 months	139 (26%)
12–24 months	61 (12%)
over 24 months	34 (6.5%)
Side of the symptoms	
Left	288 (55%)
Right	223 (43%)
Both	10 (1.9%)
Median follow period (years, IQR)	18.3 (6.5)

^1^Information on preoperative smoking was available for 372 patients

^2^Weight and height were available for 501 patients

### Data collection from medical records

Helsinki University Hospital is a part of the Hospital District of Helsinki and Uusimaa (HUS). It provides health care services for the population of over *circa* 1.6 million people and consists of 22 hospitals. These hospitals share the same electronic medical records.

The medical records of the initial visit during the index surgery were used to confirm the patient’s eligibility to the study and to collect baseline, operative, and follow-up data. The patient’s discharge condition was retrospectively evaluated/rated based on the medical records on a 5-point Likert scale (5, substantially better; 4, slightly better; 3, the same; 2, slightly worse; and 1, substantially worse) [11] by study personnel. Follow-up visits were usually performed at 1 to 2 months after surgery. Reports of these visits were used to rate symptom relief and to evaluate the patient’s condition similarly along a 5-point Likert scale. Possible postoperative complications from the follow-up period were recorded.

Each patient’s later medical records were examined (data collection in June 2018) to determine if further lumbar spine surgery procedures had been performed or if the patient had been referred for consideration of new surgeries.

### Data collection from questionnaires

In Finland, each person is granted a personal identification number at birth or on becoming a permanent resident. The identification number is used when the person is in contact with the healthcare system. With this number, healthcare organizations can request the patient’s contact address from the Population Register Center. Those patients who had an address in Finland were then sent a letter containing information regarding the study, an informed consent form, an ODI questionnaire, and a general questionnaire. Those who did not respond to the letter were sent one reminder.

The ODI questionnaire was utilized to assess the extent of disability caused by lower back pain (range 0 to 100, with a higher score indicating greater disability). The general questionnaire contained questions regarding patients’ working situations, smoking status before surgery, the patient’s satisfaction with their surgery at the present time, how many and what kind of lumbar spine surgeries the patient had undergone after the index surgery, and when the operations were performed. The information on the number of lumbar spine operations was used to complete the data collected from the medical records (the questionnaire provided additional information on the additional surgeries for 2.7% of the responding patients).

### Statistical methods

The statistical analyses were conducted with R, version 4.1.0 (R Core Team 2021). Dependent continuous variables (non-normally distributed) and ordinal variables were analyzed with the Mann–Whitney *U* and Kruskal–Wallis tests. Spearman’s rank correlation test was utilized to measure the correlation between the variables. The categorical variables between the subgroups were analyzed with Fisher’s exact and chi-square tests. For large contingency tables, the Pearson chi-square test was used if the assumptions could be met; otherwise, the maximum likelihood ratio chi-square test was used. Paired categorical variables were analyzed with the McNemar test. The differences in the Kaplan–Meier analyses of time to event between the subgroups were compared with the results of a log-rank test. A *p* value smaller than 0.05 was considered significant.

## Results

### Surgeries

Microdiscectomy was the most common surgical procedure conducted (96%), and one of the two lowest lumbar spine levels was the target in over 95% of the surgeries. The most common complication was an incidental durotomy in 4.0% of patients (Table 2). In total, 7 patients (1.3%) developed new neurological symptoms after surgery, which were still present at the clinical follow-up. Two patients (0.4%) had postoperative superficial wound infection. No deep surgical infections or postoperative hematomas were encountered.

**Table 2:** Operative data

	Number (%)
Number of patients	526
Surgical technique	
Microdiscectomy	503 (96%)
Microdiscectomy and microdecompression	16 (3.0%)
Microdecompression	7 (1.3%)
Emergency surgery^1^	77 (15%)
Duration of the surgery	
(Median, min–max)	45 (15–208)
Operated intervertebral disc level	
L5–S1	262 (50%)
L4–L5	242 (46%)
L3–L4	14 (2.7%)
L2–L3	0 (0%)
L1–L2	1 (0.2%)
Two levels	7 (1.3%)
Postoperative days spent in the hospital	
0–1	389 (74%)
2–3	121 (23%)
4 +	15 (2.9%)
Complications^2^	
Dural tear	21 (4.2%)
New sensory symptom	4 (0.8%)
New motor symptom	2 (0.4%)
Neuropathic pain	1 (0.2%)
Wound infection	2 (0.4%)
Hematoma	0 (0.0%)
Other^3^	3 (0.6%)

^1^Indicated by unbearable pain, severe motor paresis, or cauda equina syndrome

^2^Surgical reports were only available for 501 patients; thus, dural tear was only possible to address for those patients, and the rest of values were counted for the whole patient population (526 patients)

^3^Two wrong level surgeries and, one case of postoperative bladder dysfunction and saddle area sensory defect

### Clinical short-term follow-up and new referrals

**Fig. 2:**
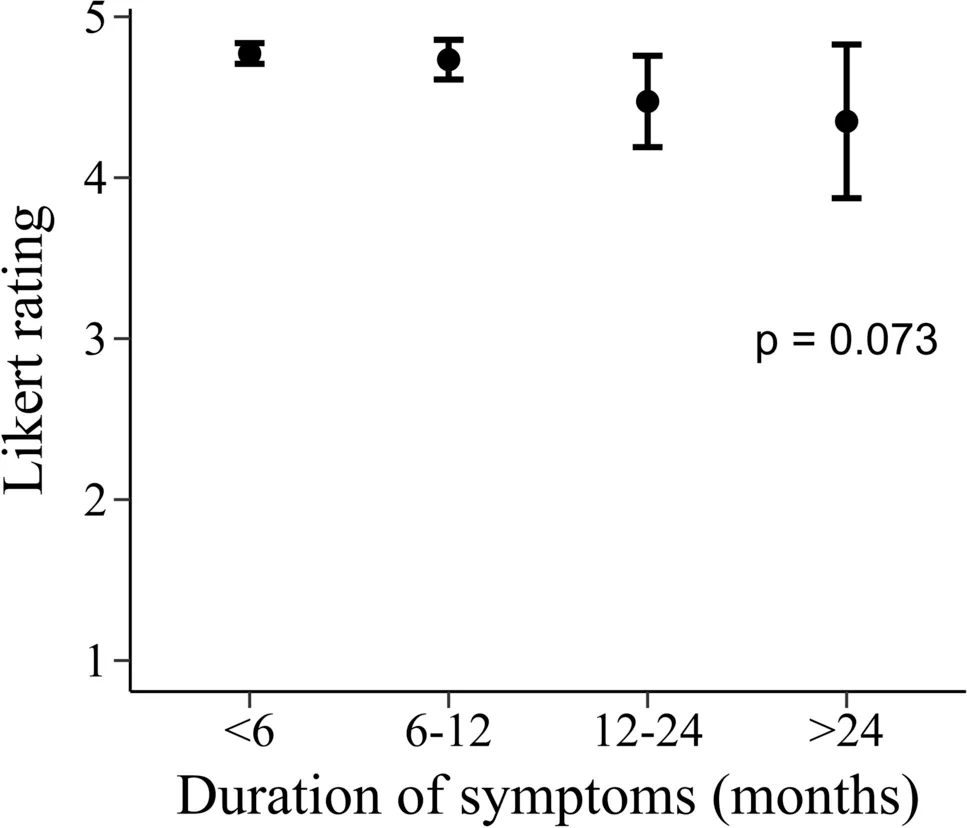
Clinical benefit as evaluated on a 5-point Likert rating at the clinical follow-up visit a median 50 days after surgery relative to the preoperative duration of symptoms (5, substantially better; 4, slightly better; 3, the same; 2, slightly worse; and 1, substantially worse). The subgroups were under 6 months (*n* = 184), 6–12 months (*n* = 90), 12–24 months (*n* = 38), and over 24 months (*n* = 20). The dots indicate the mean Likert scores for the subgroups. The error bars display the 95% confidence interval for the means. The correlation between the variables was analyzed with the Spearman’s rank correlation test

Out of the 526 patients, 330 (63%) patients arrived at a routinely scheduled clinical follow-up appointment at a median of 50 days after surgery. The most common reason for not attending the follow-up visit was the patient’s request to schedule it at a different hospital or clinic. Review of the clinical reports showed that a total of 96% of the patients improved after surgery (Table 3). Age (*p* = 0.77), gender (*p* = 0.87), or smoking status (*p* = 0.42) did not affect the patient’s condition in the clinical short-term follow-up. There was a trend toward worse condition for patients with BMI over 25 (*p* = 0.059), and patients with longer preoperative duration of symptoms (*p* = 0.073) (Fig. 2).

**Table 3:** Clinical benefit as evaluated on 5-point Likert scale^1^ at clinical follow-up of a median 50 (7–218) days after surgery

Likert scale value	1 (*N*, %)	2 (*N*, %)	3 (*N*, %)	4 (*N*, %)	5 (*N*, %)	*p*-value^2^
All patients	2 (0.6%)	2 (0.6%)	10 (3.0%)	65 (20%)	253 (76%)	
Gender						0.87
Male	1 (0.5%)	2 (1.0%)	5 (2.5%)	39 (20%)	153 (77%)	
Female	1 (0.8%)	0 (0%)	5 (3.8%)	26 (20%)	100 (76%)	
Age at the time of the surgery						0.77
Over 30	2 (0.9%)	1 (0.4%)	6 (2.6%)	44 (19%)	174 (77%)	
Under 30	0 (0%)	1 (1.0%)	4 (3.8%)	21 (20%)	79 (75%)	
Smoking at the time of the surgery						0.42
Yes	0 (0%)	0 (0%)	1 (1.1%)	24 (26%)	69 (73%)	
No	1 (0.6%)	2 (1.3%)	5 (3.2%)	25 (16%)	124 (79%)	
Body mass index						0.059
Over 25	2 (1.6%)	1 (0.8%)	5 (3.9%)	29 (23%)	92 (71%)	
Under 25	0 (0%)	1 (0.5%)	4 (2.1%)	33 (17%)	152 (80%)	

^1^Patient’s condition at the clinical follow-up visit was retrospectively evaluated based on medical records on a 5-point Likert rating scale (5, substantially better; 4, slightly better; 3, the same; 2, slightly worse; and 1, substantially worse)

^2^Mann–Whitney *U* test

**Fig. 3:**
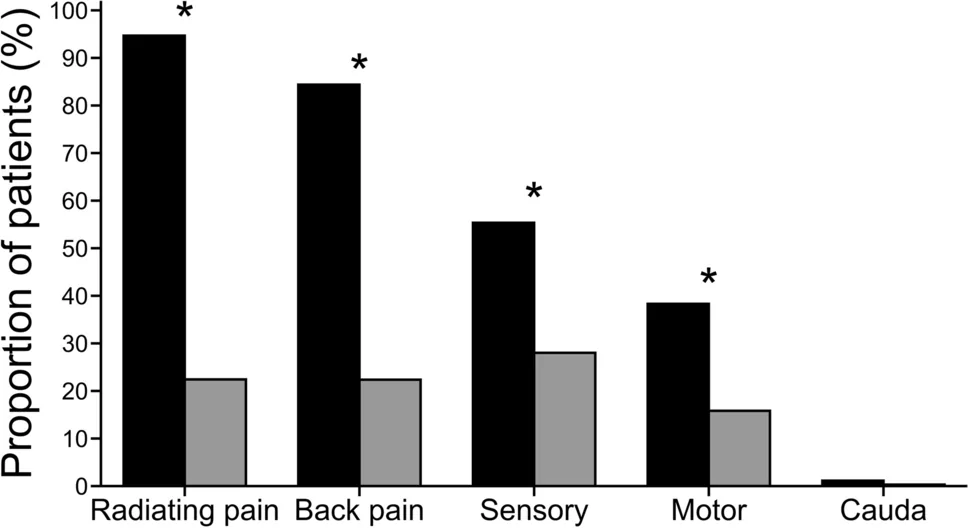
Proportion of patients suffering from symptom types before surgery (*n* = 526) and at clinical follow-up (*n* = 330). Black bars represent the symptoms patient had before surgery, and the white bars indicate the symptoms patient still had (to any mentioned extent, even if milder in intensity) at the clinical follow-up visit a median 50 days after surgery. Radiating pain, pain radiating to the lower limb(s); sensory, sensory disturbance in lower limb(s); motor, motor defect in lower limb(s); and cauda, cauda equina syndrome. A McNemar test was conducted to test for significance. *p < 0.05

The frequency of reporting all of the different types of symptoms was reduced from the preoperative state to the clinical follow-up (Fig. 3). Specifically, there was a reduction in the proportion of patients reporting back pain (84% vs 22%, *p* < 0.001), radiating leg pain (95% vs 22%, *p* < 0.001), sensory symptoms (55% vs 28%, *p* < 0.001), motor symptoms (38% vs 16%, *p* < 0.001), and cauda syndrome (1.1% vs 0.3%, *p* = 0.25).

Out of all 526 patients, 357 patients (68%) did not have any new referrals for evaluation of surgical treatment for degenerative lower spine disorders to any of the HUS hospitals during the follow-up time (Table 4). A total of 119 patients (23%) had one referral, 32 (6.1%) had two referrals, and 18 (3.4%) had three or more referrals.

**Table 4:** Follow-up, reoperations, and further surgeries

	Number (%)
Number of patients	526
Participated in clinical follow-up^1^	330 (63%)
Median time to follow-up (days)	50
New referrals^2^	
0	357 (68%)
1	119 (23%)
2	32 (6.1%)
3	9 (1.7%)
4	7 (1.3%)
5 +	2 (0.4%)
Reoperation in under 30 days	21 (4.0%)
Another lumbar surgery during follow-up period^3^	136 (26%)
Same level as index surgery^4^	111 (76%)
Type of first lumbar surgery after index surgery	
Microdiscectomy	114 (76%)
Microdiscectomy and microdecompression	13 (8.7%)
Microdecompression	10 (6.7%)
Lumbar fusion	8 (5.3%)
Spinal cord stimulator installment	2 (1.3%)
Exploration	1 (0.7%)
Percutaneus nucleotomy	1 (0.7%)
Unknown	1 (0.7%)
Number of lumbar spine operations after index surgery	
0	376 (72%)
1	107 (20%)
2	26 (5%)
3	9 (1.7%)
4	5 (1.0%)
5 or more	3 (0.6%)
Cervical spine surgery during follow-up	13 (2.5%)
Lumbar fusion surgery during follow-up^5^	18 (3.4%)
Spinal cord stimulator installment during follow-up^5^	6 (1.1%)

^1^The most frequent reason for not participating in the clinical follow-up visit was it being scheduled at a different hospital or clinic

^2^New referrals to evaluate the need of new surgical treatment for degenerative lumbar spine disorder

^3^Reoperations during the first 30 days of follow-up time excluded

^4^Not including patients whose surgery was spinal stimulator installment, or the level of the surgery was unknown

^5^Lumbar fusion or epidural pain stimulator installment at any time point during the follow-up period

### Early reoperations

Twenty-one patients had an additional surgery of the lumbar spine within 30 days of the index surgery (i.e., an early reoperation; Table 4). Of these surgeries, 18 (86%) were performed on the same intervertebral level as the index surgery. The type of surgery was a microdiscectomy in 17 cases, a microdecompression in two cases, a combination of microdiscectomy with microdecompression in one case, and an exploration in one case.

Patients older than 30 at the time of the surgery (5.0% vs 1.8%, *p* = 0.096) and patients with a BMI over 25 (5.9% vs 2.7%, *p* = 0.10) had a trend toward more frequent early reoperations. Smoking status (3.8% vs 4.2%, *p* = 1), and male gender (3.8% vs 4.3% *p* = 0.82) did not affect the risk of early reoperation.

### Further lumbar surgeries

Excluding the aforementioned reoperations during the first 30 days, 136 patients (26%) had one or more new lumbar spine surgeries in the 18.3-year median follow-up time that involved the index or another level (Table 4). Therefore, the annual risk of new lumbar surgery was 1.4%. Age over 30 at the time of the surgery (24% vs 30%, *p* = 0.25), male gender (27% vs 24%, *p* = 0.36), smoking status (30% vs 25%, *p* = 0.33), or a BMI over 25 (27% vs 26%, *p* = 0.84) did not affect the risk of further lumbar surgeries. There was also no significant difference in further surgery rates for the patients who participated in the follow-up visit (28% vs. 22%, *p* = 0.15).

Eighteen patients (3.4%) had a lumbar fusion and six patients (1.1%) underwent a trial implantation or permanent implantation surgery for a spinal cord stimulator (SCS) during the follow-up. Additionally, 13 patients (2.5%) had undergone cervical spine surgery during the follow-up period for degenerative disc disorder.

**Fig. 4:**
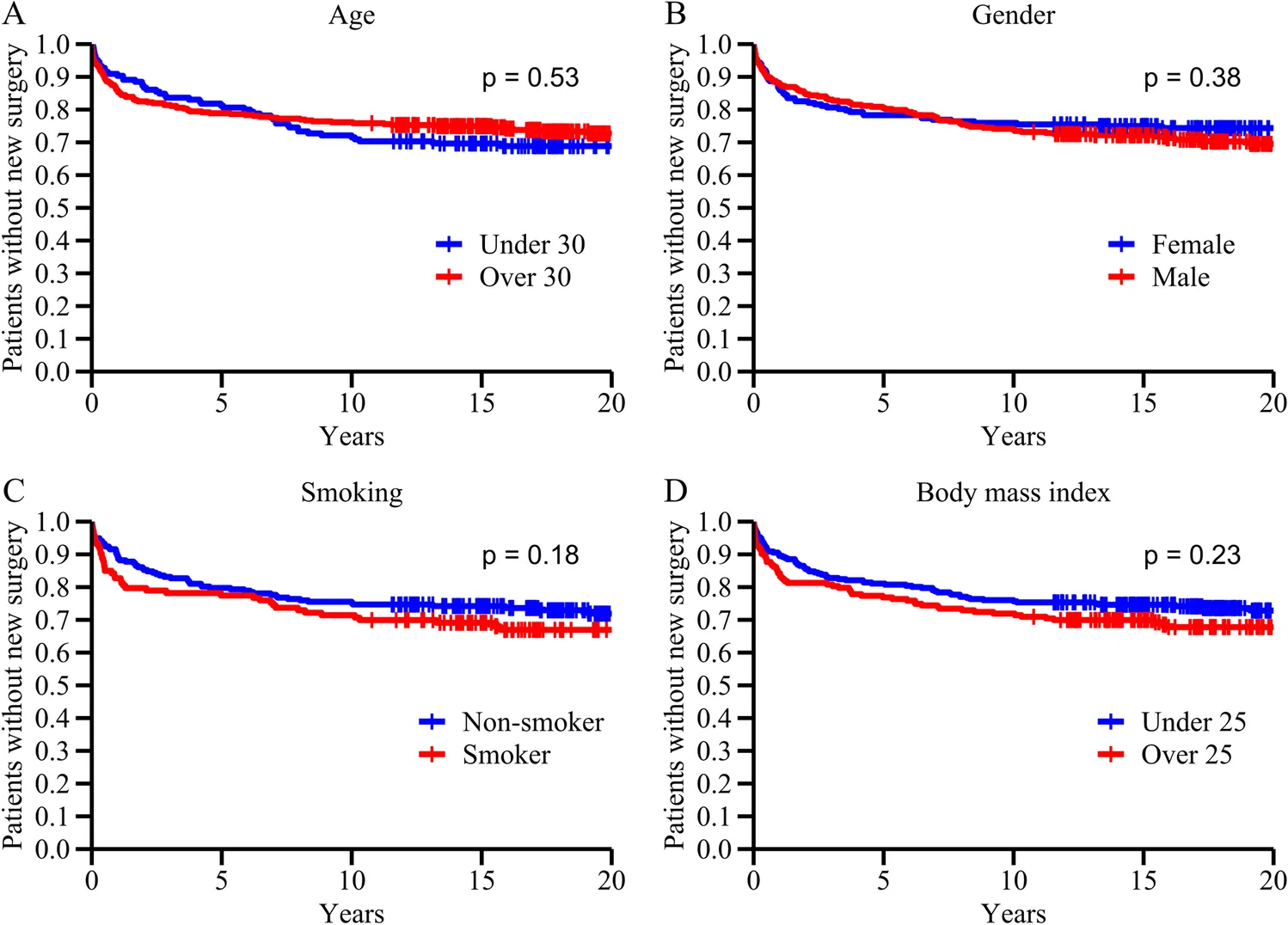
Time to new lumbar surgery for different subgroups. Kaplan–Meier plots represent the proportion of patients without need of new surgery as a function of time as years from the index surgery. The subgroups were compared to each other with the log-rank test. **A** Includes patients who were over 30 years old at the time of the surgery (red *n* = 360) and patients who were under 30 years of age (blue, *n* = 165); **B** female patients (blue, *n* = 212) and male patients (red, *n* = 313); **C** patients who were smokers (red, *n* = 133) and non-smokers (blue, *n* = 237); and **D** patients with a body mass index under 25 (blue, *n* = 296) and over 25 (red, *n* = 203. In total, 11 patients had new surgery during follow-up period, but the exact date of surgery was unknown. The year or year and month of the new surgery was known for 10 patients. These patients were included in the analysis by inputting the median date as the approximal date of the surgery. The patient with no information on the date of the new surgery was excluded from the analysis

The Kaplan–Meier mean estimate for the time to new surgery was 20.4 years for all patients. Male gender, age over 30, BMI over 25, and smoking status did not affect the time to further surgery (Fig. 4).

### General survey answers

Out of the 526 patients, 317 (60%) answered the study questionnaires that were sent out now at median 18.3 years after their index surgery (Table 5). The median age of the responding patients was 53. Characteristics associated with higher response rates were older age (*p* < 0.001), female gender (*p* = 0.001), non-smoking status (*p* = 0.008), and lower BMI (*p* = 0.001). There was no statistical difference in further lumbar surgeries between patients who responded to the questionnaires and non-responders (25% vs. 27%, *p* = 0.54).

**Table 5:** Working status, smoking status, and satisfaction on surgery

	Number (%)
Number of patients	526
Replied to survey	317 (60%)
Median age (years, IQR)	53.7 (8.6)
Working status	
Working	282 (89%)
Jobless	10 (3.2%)
Full time disability pension	14 (4.4%)
Part time disability pension	5 (1.6%)
Old-age pension	5 (1.6%)
Other	1 (0.3%)
Disability or part time disability pension due to	
Lower spine disorder	7 (37%)
Other disorder	12 (63%)
Smoking	
Currently	46 (15%)
Before surgery	97 (32%)
Satisfaction on surgery	
Very satisfied	217 (69%)
Satisfied	67 (21%)
Unable to say	12 (3.8%)
Dissatisfied	12 (3.8%)
Very dissatisfied	8 (2.5%)
Would you choose surgery again?	
Yes	295 (95%)

Overall, patients were satisfied with their index treatment, with 90% reporting either being very satisfied or satisfied, and 95% indicating that they would choose the same treatment again.

A total of 282 patients (89%) reported being employed when they responded to the questionnaire, which was consistent with the findings in 2014 indicating that 86% of the Finnish 45 to 54-year-old population was employed. Ten patients (3.2%) reported being unemployed. The number of patients on either part-time or full-time disability pension was 19 (6.0%), of which 7 cases (39%) were attributed to lumbar spine disorders.

### Oswestry Disability Index (ODI) scores

The mean ODI score was 8.1 at the median of 18.3 years after index surgery (Table 6). Females had higher mean ODI scores than men (10.3 vs 6.3, *p* < 0.001). An age under 30, BMI over 25 or smoking status did not affect the ODI score. Patients who had undergone another lumbar spine surgery after the index surgery had higher scores than those who had not (12.2 vs 6.6, *p* < 0.001). Additionally, there was a trend of a longer duration of symptoms prior to surgery correlating with deteriorating ODI scores (*p* = 0.10) (Table 6).

**Table 6:** Oswestry Disability Index (ODI) scores

Variable	*N*	Mean score	*p*-value^1^
All patients	316	8.1	
Gender			< 0.001
Male	170	6.3	
Female	146	10.3	
Age at the time of the surgery			0.49
Over 30	228	8.5	
Under 30	88	7.3	
Smoking at the time of the surgery			0.30
Yes	97	7.1	
No	209	8.5	
Body mass index			0.77
Over 25	110	8.7	
Under 25	192	7.8	
Duration of symptoms before surgery			0.10
Under 6 months	174	7.0	
6–12 months	87	7.3	
12–24 months	34	11.1	
Over 24 months	21	16.2	
Further lumbar surgery during follow-up period			
Yes	86	12.2	< 0.001
No	230	6.6	
Number of further lumbar surgeries			< 0.001
0	230	6.6	
1	67	10.9	
2	11	11.4	
3	8	24.0	
Lumbar fusion during follow-up period			
Yes	6	35.3	< 0.001
No	310	7.6	
Spinal stimulator during follow-up period			0.004
Yes	3	42.0	
No	313	8.6	

316 (60%) patients completed the ODI questionnaire a median of 18.3 years after index surgery

^1^Mann–Whitney *U* test or Spearman’s rank correlation test

An increasing number of further lumbar spine surgeries were significantly correlated with higher ODI scores. The mean score for patients having no further surgery was 6.6, while it was 10.9 for those with one further lumbar surgery, and 11.4 for those with two further surgeries, and 24.0 for those with three or more further surgeries (*p* < 0.001).

The respondents of the survey included six patients who had a lumbar fusion performed during the follow-up period. Their mean ODI score was 35.3, and for other patients, the mean score was 7.6 (*p* < 0.001). An SCS had been installed for at least a trial period in three patients in the responding sample. Their mean score was 41.0, while it was 7.8 for the rest of the patients (*p* = 0.004).

## Discussion

There is a lack of studies on the outcome for young adult patients after surgery for lumbar disc herniation. Young patients have long expected life, and a poor long-term outcome could have negative consequences for these patients. Our study shows that both the short-term and the long-term outcomes are favorable for this patient group. Vast majority patients showed excellent short-term recovery from their symptoms and in the long term experienced a positive outcome, which was indicated by the patient cohort’s mean ODI score and employment status being comparable with those of the normative population. Unfortunately, a minority of patients who underwent numerous lumbar spine surgeries presented a notably worse outcome and increased disability.

In total, 26% of patients underwent another lumbar surgery during the median 18.3-year follow-up period. This seemingly high rate of new surgeries reflects the long follow-up period, as in the Maine Lumbar Spine Study, 25% of patients had undergone a new lumbar spine operation within only a 10-year follow-up period [1], and in the Spine Patient Outcomes Research Trial, the reoperation rate was 15% in only 8 years [10].

The annual estimated rate of lumbar disc surgery in our patient cohort was 1700 per 100,000 people (167 surgeries in 18.3 follow-up years), which was higher than that of the general Finnish population (80/100,000 people) in 1994 [8]. The incidence for the general population, however, dates back to the Keskimäki study from the 1990s, and it is likely that diagnostics and availability of surgery have been lower at that time. Nevertheless, our result is in accordance with an earlier study that reported a ten times higher rate of lumbar disc surgery for individuals with prior lumbar disc surgery than for the general population [2].

In the present study, the patients reported a low level of back-related disability, which was demonstrated by the low mean ODI score of 8.1. The patient cohort’s median age at the time of data collection was 54 years, and the previously reported normative ODI score for the 50–59 age group was 9.05 [15]. Accordingly, the mean ODI score of this group is comparable to that of the normative population. Patients who underwent further lumbar surgery and lumbar fusion or had an implantation of a SCS device had significantly higher scores than the other patients, and an increasing number of lifetime lumbar surgeries were correlated with deteriorating ODI scores. These results are consistent with multiple earlier studies reporting that repeated lumbar surgery is associated with worse ODI scores [5, 10]. Nevertheless, this cannot be interpreted merely as indicating that an SCS or lumbar fusion would undermine the patient’s outcome; rather, it reflects the condition of the patients requiring these surgical procedures.

Another factor indicating a satisfactory functional long-term outcome was the similarity of the patients’ working status to that of the general population in Finland. In our patient sample (median age of 54 years at the end of follow-up), the employment rate was 89%. In the age group of 45–54 in Finland, 85% of males and 87% of females were working in 2014 [6]; therefore, the employment rate of the patients was comparable with that of the general population. Furthermore, 19 patients (6.0%) were on disability pension, and this is tolerable since disability pension rates for the general population in the aforementioned age group was 6% for males and 5% for females [6]. However, seven patients (2.2%) were on disability pension due to lower spine disorder.

Hence, patients who underwent repeated lumbar spine surgeries during the follow-up period had a worse long-term outcome than other patients, which highlights the importance of understanding the risk factors for further surgery. None of the baseline characteristics presented as significant risk factors for further surgery, and in contrast to the findings of a recent meta-analysis on risk factors for reoperation [7], smokers did not have a higher rate of further surgery than non-smokers in this study. This result could be explained by 61% of the patients quitting smoking during the follow-up period, which could have influenced the long-term results and diluted the effects of smoking.

The study has several limitations. First, survey studies have a selection bias due to respondents differing from the non-respondents. Even though we observed a satisfactory response rate of 60% at 18 years after index surgery, the responding patients were more likely to be older, female, non-smokers, and have a lower initial BMI than the non-responding patients. These differences could have influenced the observed ODI scores and reported working status of the patient cohort. However, we analyzed the further surgery rates for patients who did not respond to the questionnaires and patients who did not participate in the clinical follow-up visit and there was no difference in the rates. Second, information on additional lumbar surgery was primarily collected from the medical records of HUS, and the information from the questionnaires was utilized to complete the data. The responses were the key method to gain information on the additional surgeries conducted outside the HUS area. Hence, it is probable that some non-responders had additional lumbar surgery outside the HUS area, leaving some additional surgeries unaccounted for. However, only 2.5% of the patient answers provided additional information on further surgeries. Assuming the extent of missing information for the non-responding patients would have been the same, receiving responses from all non-responders would have given us insight into only five more further surgeries. Therefore, we cautiously suggest that the effect of this bias is minor.

## Conclusions

In conclusion, patients who underwent surgery for lumbar disc herniation in their early adulthood experienced, as expected, good to excellent short-term recovery from their symptoms. In the long term, a notable proportion of the patients required additional lumbar surgery during the follow-up period; however, the ODI scores were comparable with those of the normative population. An increasing number of lumbar surgeries were correlated with deteriorating outcomes measured on the ODI. Further research on the risk factors is needed to describe with greater precision which patients are at higher risk of additional surgery and how to possibly prevent dissatisfactory outcomes.

## Data Availability

The datasets generated during and/or analyzed during the current study are available from the corresponding author on reasonable request.
